# *Plasmodium falciparum* EPCR-binding PfEMP1 expression increases with malaria disease severity and is elevated in retinopathy negative cerebral malaria

**DOI:** 10.1186/s12916-017-0945-y

**Published:** 2017-10-13

**Authors:** Estela Shabani, Benjamin Hanisch, Robert O. Opoka, Thomas Lavstsen, Chandy C. John

**Affiliations:** 1 Ryan White Center for Pediatric Infectious Diseases and Global Health, Indiana University, 1044 W Walnut St R4 402D, Indianapolis, Indiana USA; 2 Department of Pediatrics, Division of Global Pediatrics, University of Minnesota, Minneapolis, Minnesota USA; 3 Children’s National Medical Center, Washington, DC USA; 4 Department of Pediatrics and Child Health, Makerere University School of Medicine, Kampala, Uganda; 5 Centre for Medical Parasitology, Department of International Health, Immunology and Microbiology, University of Copenhagen and Department of Infectious Diseases, Copenhagen, Denmark

**Keywords:** Severe malaria, Cerebral malaria, Retinopathy, *Plasmodium falciparum* erythrocyte membrane protein 1 (PfEMP1), Transcript levels

## Abstract

**Background:**

Expression of group A and the A-like subset of group B *Plasmodium falciparum* erythrocyte membrane protein 1 (PfEMP1) is associated with severe malaria (SM). The diversity of *var* sequences combined with the challenges of distinct classification of patient pathologies has made studying the role of distinct PfEMP1 variants on malaria disease severity challenging. The application of retinopathy in the recent years has provided a further method to clinically evaluate children with cerebral malaria (CM). The question of whether children with clinical CM but no retinopathy represent a completely different disease process or a subgroup within the spectrum of CM remains an important question in malaria. In the current study, we use newly designed primer sets with the best coverage to date in a large cohort of children with SM to determine the role of *var* genes in malaria disease severity and especially CM as discriminated by retinopathy.

**Methods:**

We performed qRT-PCR targeting the different subsets of these *var* genes on samples from Ugandan children with CM (*n* = 98, of whom 50 had malarial retinopathy [RP] and 47 did not [RN]), severe malarial anemia (SMA, *n* = 47), and asymptomatic parasitemia (AP, *n* = 14). The primers used in this study were designed based on *var* sequences from 226 Illumina whole genome sequenced *P. falciparum* field isolates.

**Results:**

Increasing severity of illness was associated with increasing levels of endothelial protein C receptor (EPCR)-binding PfEMP1. EPCR-binding PfEMP1 transcript levels were highest in children with combined CM and SMA and then decreased by level of disease severity: RP CM > RN CM > SMA > AP.

**Conclusions:**

The study findings indicate that PfEMP1 binding to EPCR is important in the pathogenesis of SM, including RN CM, and suggest that increased expression of EPCR-binding PfEMP1 is associated with progressively more severe disease. Agents that block EPCR-binding of PfEMP1 could provide novel interventions to prevent or decrease disease severity in malaria.

**Electronic supplementary material:**

The online version of this article (doi:10.1186/s12916-017-0945-y) contains supplementary material, which is available to authorized users.

## Background

Cerebral malaria (CM) and severe malarial anemia (SMA) are the main drivers of morbidity and mortality due to *Plasmodium falciparum.* CM is characterized by coma and has a mortality rate of 13–15% [1, 2]. In CM, adhesion of infected erythrocytes (IEs) to other uninfected erythrocytes (UEs) (rosette formation) and sequestration of IEs, leukocytes, and platelets to the blood-brain barrier (BBB) endothelium, combined with an imbalanced immune response and endothelium activation are thought to lead to BBB dysfunction and adverse clinical outcomes [3–7]. Within CM, malarial retinopathy has been proposed to distinguish “true” CM (retinopathy positive, RP) from coma due to other causes, with incidental *P. falciparum* parasitemia (retinopathy negative, RN) [8]. However, it has been suggested that RN CM may be part of the clinical spectrum of CM [9]. Assessment of parasite gene expression could help determine whether parasite virulence factors expressed in RP CM are also associated with RN CM.

SMA is characterized by severe anemia and inflicts a substantial burden in sub-Saharan Africa, causing 20% of *P. falciparum* hospitalizations [10]. In settings where there is easier access to blood transfusions, the mortality from SMA is lower than that from CM (1–5%) [1, 11]. In SMA, destruction of IEs and UEs, dyserythropoiesis, and suppression of erythropoiesis are considered important contributors to severe anemia. Little is known about how parasite virulence factors contribute to the development of these different clinical manifestations of severe malaria.

*P. falciparum* erythrocyte membrane protein 1 (PfEMP1) is considered a key virulence factor in malaria, as it binds to various host receptors on the endothelium or UEs (rosetting) to sequester infected erythrocytes from circulation and destruction in the spleen [12–17]. PfEMP1 is a target of antibody-mediated immunity [18], and in response, PfEMP1 molecules have diversified extensively. Despite this extensive sequence variation, PfEMP1 function is conserved, and PfEMP1 molecules have a highly ordered domain composition, kept in check by highly ordered organization and mechanism of recombination of the encoding *var* genes [19–21]. Thus, each haploid parasite genome carries 50–60 polymorphic *var* genes [14, 22, 23], divided by chromosomal location and direction of transcription into groups A, B, and C. The extracellular portion of PfEMP1 varies in organization and length but comprises a combination of Duffy binding-like domains (DBLα-ζ) and cysteine-rich interdomain regions (CIDRα-δ) [20, 21]. The N-terminal domain composition of PfEMP1 is conserved and linked to the genetic control of *var* groups. Group A *var* genes encode PfEMP1 with CIDRα1 domains shown to bind endothelial protein C receptor (EPCR) [24] or a set of more diverse CIDRβ/γ/δ domains of unknown function, but potentially associated with rosetting [16]. Groups B and C *var* genes encode cluster of differentiation 36 (CD36)-binding PfEMP1 [25]. One exception to this rule is the so-called conserved tandem arrangements known as domain cassette 8 (DC8) PfEMP1 [21], which is a group A-like EPCR-binding PfEMP1, recombined into a group B *var* gene location.

Consensus from previous studies of *var* gene expression in patients shows that expression of group A and DC8 *var* genes is associated with severe malaria [26–32]. Specifically, group A and DC8 PfEMP1 that bind EPCR have been suggested to play a key role in severe malaria, through their ability to support IE binding to various microvasculature beds [33, 34] and through reducing the production and cytoprotective effects of activated protein C, due to functional impairment of EPCR upon PfEMP1 engagement [35–37]. As a result, the extent of PfEMP1-EPCR binding could determine the amount of sequestration, coagulation defects, endothelial activation, and permeability, which in turn could define the outcomes of severe malaria. In line with this, EPCR-binding PfEMP1 transcript levels were recently associated with increased disease severity, from asymptomatic infections to both SMA and CM, in Tanzanian children [32]. More studies are needed to confirm these findings. In particular, the importance of EPCR-binding PfEMP1 in RP vs. RN CM is not well understood.

In the current study, we used qRT-PCR primers with coverage and high specificity [32] for group A and DC8 *var* genes to assess differential gene expression in parasites from Ugandan children with CM vs. SMA, in children with CM with vs. without retinopathy, and in children with CM who died vs. those who survived. The primers used in this study have been recently designed [32] based on the analysis of 226 *var* genomes as compared to only 7 used by the previous studies in the field [31, 38]. As a result, these primers provide the best coverage to date, and the current study presents the first time they are used to study the association of *var* types with CM discriminated by retinopathy.

## Methods

### Study design

This prospective cohort study with the overall goal of understanding the effects of severe malaria on neurodevelopment was conducted at Mulago National Referral and Teaching Hospital in Kampala, Uganda in 2008–2015 and enrolled children with CM, children with SMA, and community children (CC). The study was reviewed and approved by the Ugandan National Council for Science and Technology (UNCST), the Makerere University School of Medicine Research and Ethics Committee, and the University of Minnesota Institutional Review Board. Written informed consent was obtained from parents or guardians of study participants.

Children between 18 months and 12 years of age, meeting the World Health Organization definition for CM or SMA, were recruited from the Acute Care Unit at Mulago Hospital as previously described [1]. CM was defined as (1) coma (Blantyre coma score [BCS] ≤ 2), (2) *P. falciparum* on blood smear, and (3) no other known cause of coma. SMA was defined as presence of *P. falciparum* on blood smear in children with hemoglobin < 5 g/dL. Exclusion criteria for children with SMA included any impairment of consciousness or having > 1 seizure. Children with severe malaria were managed according to the Ugandan Ministry of Health treatment guidelines at the time, which included quinine treatment [1].

CC were recruited from the nuclear family, extended family, or household compound area of children with CM or SMA. Eligible CC were aged 18 months to 12 years and currently healthy. A blood smear was taken from these children at the time of enrollment, and those who had any density of P. falciparum on the smear are indicated here as asymptomatic parasitemic (AP). Exclusion criteria for all children included (1) known chronic illness requiring medical care, (2) known developmental delay, or (3) prior history of coma, head trauma, cerebral palsy, or hospitalization for malnutrition. A total of 269 children with CM, 232 children with SMA, and 217 CC were enrolled in the study. Of the 217 CC, 32 had asymptomatic parasitemia.

### Sample collection and RNA isolation

Whole blood was collected at enrollment in PAXgene Blood RNA preservative solution (PreAnalytiX, Hombrechtikon, Switzerland) in a ratio of 2.76 mL of additive per mL of blood. The samples were stored long term at –80 °C. RNA was isolated using the PAXgene Blood RNA Kit (PreAnalytiX, Hombrechtikon, Switzerland).

### Primer design

Primers were designed and optimized and previously described [32]. Briefly, the primers used in this study were designed based on full-length DBL and CIDR domain encoding sequences from seven *P. falciparum* genomes and 226 Illumina whole genome sequenced *P. falciparum* field isolates [32]. Primer sequences, coverage, and specificity are depicted in Additional file 1: Figure S1.

### Quantification of *var* transcript levels by qRT-PCR

Total RNA was treated with DNase I (Invitrogen, Carlsbad, CA, USA). Complementary DNA (cDNA) was synthesized using random hexamers and the SuperScript® III First-Strand Synthesis System (Invitrogen, Carlsbad, CA, USA) according to manufacturer’s instructions. qRT-PCR was performed in 20-μL reactions using KiCqStart® SYBR® Green qPCR ReadyMix™ (Sigma-Aldrich, St. Louis, MO, USA) with the 7500 Real Time PCR System (Applied Biosystems, Foster City, CA, USA). Amplification was performed following the previously published conditions [31], and data was collected at the final elongation step. No reverse transcriptase and no template controls for both housekeeping genes were included in the plates to rule out DNA contamination in the RNA samples and any nucleic acid contamination in reagents, respectively. Gene expression was normalized to the average of two housekeeping genes: seryl-tRNA synthetase and fructose-bisphosphate aldolase (ΔC_t var_primer_ = C_t var_primer_ − C_t average_control primers_). ΔC_t var_primer_ was transformed into arbitrary transcript units using T_u_ = 2^(5−ΔCt)^. Only samples that had a C_t average_control_ below 25 were included in the analysis. Melting temperature analysis was performed for each target, and only samples with *T*_m_ within 1.7 °C of median *T*_m_ were analyzed. If only primer dimers or non-specific larger targets were detected, T_u_ for that target was assigned as 1.

### Laboratory testing

Peripheral blood smears were assessed for *Plasmodium* species by microscopy with Giemsa staining using standard protocols. Blood culture was performed with the Bactec 9050 Blood Culture System (Becton Dickinson, Franklin Lakes, NJ, USA). Blood culture samples negative by this method were further cultured on blood agar or chocolate agar to further rule out bacterial infection. PfHRP-2 quantification was performed using the Malaria Ag CELISA (Cellabs, Brookvale, Australia). Sequestered parasite biomass was calculated as previously described [39]. Plasma soluble intercellular adhesion molecule-1 (sICAM-1), vascular cellular adhesion molecule-1 (sVCAM-1), and soluble P-Selectin and E-Selectin were measured by magnetic cytometric bead assay in plasma diluted 1:300 (R&D Systems, Minneapolis, MN, USA) according to manufacturer’s instructions with a BioPlex-200 system (Bio-Rad, Hercules, CA, USA). Plasma angiopoietin-2 (Ang-2) and von Willebrand factor (VWF) levels were quantified using the human angiopoietin-2 DuoSet ELISA kit (R&D Systems, Minneapolis, MN, USA) and the REAADS von Willebrand Factor activity ELISA kit (Corgenix, Broomfield, CO), respectively. Soluble EPCR levels in plasma were quantified using the Asserachrom® sEPCR immunoassay (Stago Group, Gennevilliers, France) according to manufacturer’s instructions.

### Malarial retinopathy diagnosis

Children were assessed for malarial retinopathy by indirect ophthalmoscopy. Ophthalmoscopy was done by medical officers in all CM patients on admission, and repeated every 24 h while they remained comatose. Before each examination, the pupils were dilated with sequential instillation of cyclopentolate 1% and tropicamide 1%. Using a binocular indirect ophthalmoscope, an eye exam was performed 30–60 min later. The medical officers were trained by an ophthalmologist experienced in the evaluation of malarial retinopathy. The study investigators and ophthalmologist then continued training and assessing the study medical officers for accuracy in this assessment and recording of the ophthalmoscopic finding. Children with retinopathy on any exam were classified as RP.

### Statistical analysis

Data was analyzed using Stata/SE 12.1 (StataCorp, College Station, TX, USA). Transcript abundance of *var* genes was compared between disease groups using the Mann-Whitney *U* test. Clinical and laboratory findings for children in the different disease groups were compared using the chi-squared test for categorical data and *t* tests for continuous measures. Associations between *var* types and parasite biomass, sequestered parasite load, and markers of endothelial activation and anemia were determined by Spearman’s correlation and adjusted for multiple comparisons by a Bonferroni correction. T_u_ for group A-EPCR binders was determined as the sum of [CIDRa1.4, CIDRa1.5a, CIDRa1.5b, CIDRa1.6b, and CIDRa1.7]T_u_-4; T_u_ for group B-EPCR binders was determined as the sum of [CIDRa1.1, CIDRa1.8a, and CIDRa1.8b]T_u_-2; T_u_ of CIDRα1 EPCR binders was calculated as the sum of [CIDRa1.1-CIDRa1.8b]T_u_-7.

## Results

### Characteristics of study population

We had RNA with sufficient volume and quality to quantify *P. falciparum var* transcript levels from 159 patients (98 with CM, 47 with SMA, and 14 classified as AP). Among the 98 children with CM, retinopathy testing was performed on all but one child, with 50 children malarial RP, and 47 RN. Twenty-one children with CM also met criteria for SMA (hemoglobin ≤ 5 g/dL). To analyze differences between children with CM and SMA, we assessed findings in the children with CM only (i.e., hemoglobin > 5 g/dL, *n* = 77) and compared these findings to those in children with SMA only (*n* = 47, see the following sections).

The median age of children in the study was 40.0 months ([25^th^ percentile, 75^th^ percentile], [28.7, 54.6]). Age and sex did not significantly differ between disease groups (Table 1). Parasite biomass, indicated by *P. falciparum* histidine-rich protein-2 (PfHRP-2) levels differed between disease groups (*P* < 0.0001, Table 1), being higher in CM than SMA than AP. Sequestered biomass followed the same trend (*P* < 0.0001, Table 1), confirming that while sequestration occurs commonly in *P. falciparum* infections, its magnitude differs among various manifestations of malaria. Children with CM who did not have RNA for *var* testing had lower peripheral parasite density than the ones who did (*P* = 0.04, Additional file 1: Table S1). In addition, a smaller proportion of children with SMA who did not have enough RNA for testing were male compared to those who did have sufficient and adequate quality RNA for testing. There were no other clinical differences between children who had vs. those who did not have RNA for testing in each group (Additional file 1: Table S1).

**Table 1:** Study population characteristics

	Cerebral malaria (CM) (*n* = 98)	Severe malarial anemia (SMA) (*n* = 47)	Asymptomatic *P. falciparum* parasitemia (AP) (*n* = 14)	*P* ^a^
Age (months), median (IQR)	41.5 (30.9–54.6)	33.4 (24.9–52.4)	48.5 (31.0–71.0)	0.14
Sex (male), *n* (%)	59 (60.2)	35 (74.5)	7 (50.0)	0.14
Weight-for-age z-score, mean (SD)	–1.11 (1.49) *n* = 97	–1.98 (1.39)	–0.31 (1.17)	0.0001^b^
Hemoglobin (g/dL), mean (SD)	7.07 (2.30)	3.81 (0.74)	11.2 (2.15)	<0.0001^c^
Parasite density (/μL), median (IQR)	67,010 (18,030–347,010) *n* = 96	43,880 (11,940–156,040) *n* = 46	2170 (520–11,880)	<0.0001^d^
Parasite load (PfHRP-2, ng/mL), median (IQR)	2648 (883–5150)	862 (288–2033) *n* = 46	88.8 (4.80–158) *n* = 13	<0.0001^c^
Sequestered biomass (x10^8), median (IQR)^e^	17,928 (5323–39,891) *n* = 96	6249 (1303–15,839) *n* = 45	469 (0–1309) *n* = 13	<0.0001^c^

^a^Analysis of variance (ANOVA), Tukey post hoc test adjustment for multiple comparisons with log10 transformed values for variables with no normal distribution. Chi-squared test was used for sex, with *P* < 0.017 considered significant to control for multiple comparisons.

^b^In post hoc testing, SMA differed from CM and AP

^c^In post hoc testing, all groups differed from each other

^d^In post hoc testing, CM and SMA differed from AP

^e^See Methods section for calculation of sequestered parasite biomass

*IQR* interquartile range, *SD* standard deviation

### Children with asymptomatic *P. falciparum* parasitemia had low levels of *var* transcripts encoding group A and DC8 PfEMP1 variants

**Fig. 1:**
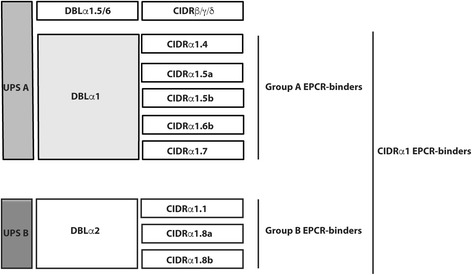
Schematics of the extracellular PfEMP1 domains, whose transcript levels are quantified in the study, and their known binding phenotype. CIDRα1-EPCR transcripts are estimated as the sum of [CIDRa1.1-CIDRa1.8b]T_u_-7; group A-EPCR transcripts are estimated as the sum of [CIDRa1.4, CIDRa1.5a, CIDRa1.5b, CIDRa1.6b, and CIDRa1.7]T_u_-4; group B-EPCR transcript levels are estimated as the sum of [CIDRa1.1, CIDRa1.8a, and CIDRa1.8b]T_u_-2. T_u_ are transcript units

The primer sets used to quantify *var* transcripts encoding different subsets of group A PfEMP1 [32] were (Fig. 1, Additional file 1: Figure S1) “DBLa1ALL”, targeting loci common to all group A *var* genes; “DBLa1.5/6/8”, targeting loci in the subset of group A genes encoding DBLα1 domains typically linked to non-EPCR-binding CIDRβ/γ/δ domains [40, 41]; and “DBLa2/1.1/2/4/7”, targeting loci common to genes encoding DBLα1 domains typically linked to EPCR-binding CIDRα1 in both groups A and B (i.e., DC8) PfEMP1. Also, primer sets specific for genes encoding CIDRδ and all EPCR-binding CIDRα1 subtypes were included, and analyzed independently as well as grouped into group A-EPCR binders (sum of [CIDRa1.4/6a, CIDRa1.5a, CIDRa1.5b, CIDRa1.6b, and CIDRa1.7]T_u_-4) and group B-EPCR binders (sum of [CIDRa1.1, CIDRa1.8a, and CIDRa1.8b]T_u_-2) (Fig. 1, Additional file 1: Figure S1). To provide an overall idea of the transcript levels of EPCR-binding CIDRα1 domains, we also determined median transcript levels of CIDRα1 EPCR-binding PfEMP1 (sum of [CIDRa1.1-CIDRa1.8b]T_u_-7).

With the exception of primer sets CIDRa1.5a, CIDRa1.5b, CIDRa1.6b, and CIDRa1.8a, median transcript levels of all *var* genes quantified in this study were higher in parasites infecting children with SM compared to AP (*P* ≤ 0.05 for all, Table 2). Only the DBLa2/1.1/2/4/7 primers reported a range of transcript abundance in AP. The DBLa2/1.1/2/4/7 primers are predicted to have a 72%/28% specificity of genes encoding EPCR/CD36-binding PfEMP1 (Additional file 1: Figure S1). As the specific primers for CIDRα1 domains showed mostly basal levels of transcripts in AP, it is therefore possible that the *var* transcripts detected in AP encoded CD36-binding PfEMP1. All AP samples included in the analysis had average C_t_ values for the two housekeeping genes below 25, which fell within the linear portion of the genomic DNA (gDNA) standard curves for both housekeeping genes (Additional file 1: Figure S2), suggesting that the observed basal expression for the rest of the *var* genes was not due to lack of sensitivity.

**Table 2:** Transcript levels of *var* domains in children with severe malaria (SM, cerebral malaria and/or severe malarial anemia) and community children who were asymptomatic (AP)

Primers	PfEMP1 group	SM (*n* = 145)	AP (*n* = 14)	*P* ^a^
CIDRa1.1	BA	8.66 (1–39.2)	1 (1–1)	0.0001
CIDRa1.8a	BA	1 (1–1.37)	1 (1–1)	0.18
CIDRa1.8b	BA	1.07 (1–3.57)	1 (1–1)	0.006
Group B-EPCR binders		13.6 (2.73–44.2)	1 (1–1.23)	<0.0001
CIDRa1.4/6a	A	4.33 (1–13.0)	1 (1–1)	0.0001
CIDRa1.5a	A	1 (1–2.10)	1 (1–1)	0.08
CIDRa1.5b	A	1 (1–1)	1 (1–1)	0.09
CIDRa1.6b	A	1 (1–1.53)	1 (1–1)	0.07
CIDRa1.7	A	2.42 (1–8.79)	1 (1–1)	0.004
Group A-EPCR binders		13.2 (3.94–34.7)	1 (1–1.06)	<0.0001
CIDRα1-EPCR binders		34.7 (8.82–84.3)	1 (1–1.99)	<0.0001
CIDR1d	A	1 (1–3.65)	1(1–1)	0.005
DBLa1ALL	A	40.2 (8.22–77.1) *n* = 144	1(1–1)	<0.0001
DBLa1.5/6/8 types	A	9.77 (3.28–21.2) *n* = 135	1(1–1) *n* = 10	<0.0001
DBLa2/1.1/2/4/7 types	A	35.6 (20.8–60.1) *n* = 134	12.9 (6.18–19.3) *n* = 10	0.006

^a^Median transcript levels were compared using Mann-Whitney *U* test

Due to the low transcript levels reported by CIDRa1.5a, CIDRa1.5b, CIDRa1.6b, CIDRa1.7, CIDRa1.8a, and CIDRa1.8b primers in both SM and AP (Table 2), results from these primers are presented as part of their larger groups (group A-, group B-, or CIDRα1-EPCR binders) rather than individually in the rest of the paper. Primers CIDRa1.1 and CIDRa1.4/6a will be presented both separately and as part of their larger subgroups (group A-, group B-, or CIDRα1-EPCR binders).

### Transcript levels of EPCR-binding PfEMP1 variants were higher in children with CM compared to those with SMA

**Fig. 2:**
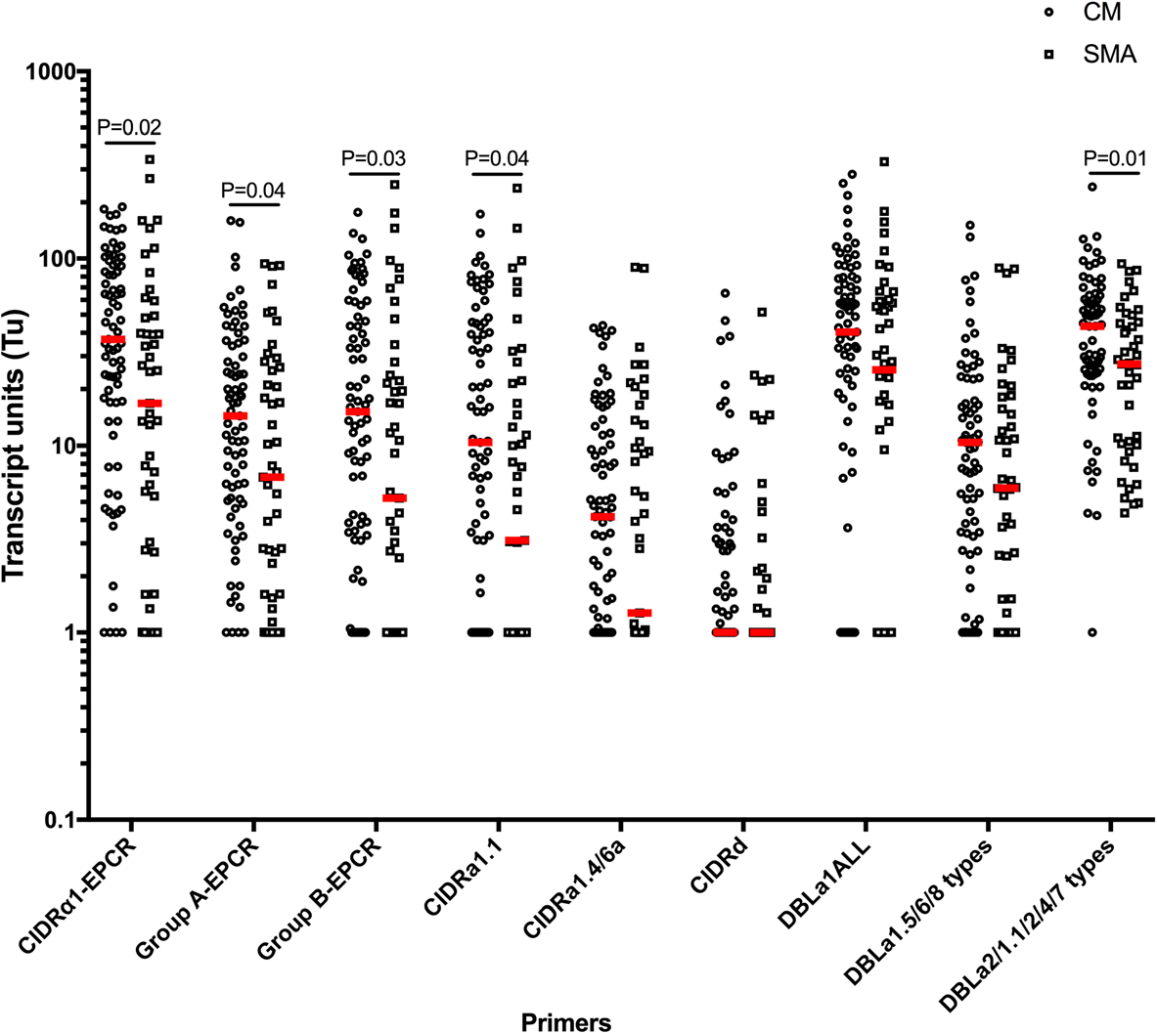
Transcript levels of EPCR-binding PfEMP1 are higher in parasites from children with cerebral malaria without severe malarial anemia than from children with severe malarial anemia. Transcript units for CIDRα1-EPCR (sum of [CIDRa1.1-CIDRa1.8b]T_u_-7), group A-EPCR (sum of [CIDRa1.4, CIDRa1.5a, CIDRa1.5b, CIDRa1.6b, and CIDRa1.7]T_u_-4), group B-EPCR (sum of [CIDRa1.1, CIDRa1.8a, and CIDRa1.8b]T_u_-2), CIDRα1.1, CIDRa1.4/6a, DBLa1ALL (all group A PfEMP1), DBLa1.5/6/8 types (group A PfEMP1 typically non-EPCR-binding), CIDRd (group A PfEMP1, non-EPCR-binding), and DBLa2/1.1/2/4/7 (group A PfEMP1, typically EPCR-binding). Transcript units of expression are shown on a logarithmic scale. The horizontal lines in red represent median values. Medians are compared by Mann-Whitney test. Cerebral malaria (*CM*, hemoglobin > 5 g/dL, *n* = 77), severe malarial anemia (*SMA*, *n* = 47)

*P. falciparum* parasites infecting children with CM and no SMA (*n* = 77) had higher median levels of *var* transcripts encoding EPCR-binding PfEMP1 (CIDRα1-EPCR, group A-EPCR, group B-EPCR, CIDRα1.1, and DBLα2/1.1/2/4/7; Fig. 2, *P* < 0.05 for all) compared to those for children with SMA. Conversely, transcript levels reported by primer sets targeting transcripts encoding the non-EPCR-binding subset of group A PfEMP1 (DBLa1ALL [*P* = 0.11], DBLa1.5/6/8 types [*P* = 0.11], and CIDR1d [*P* = 0.31]) did not differ between CM and SMA. In independent regression models, a log base 10 increase in CIDRα1-EPCR, group A-EPCR, group B-EPCR, and DBLa2/1.1/2/4/7 transcript levels were associated with increased risk of CM vs. SMA, when adjusted for PfHRP-2 levels, age, sex, and weight-for-age z-score (odds ratio [OR] 2.27, 95% confidence interval [CI] 1.21–4.27, *P* = 0.01; OR 2.64, 95% CI 1.26–5.53, *P* = 0.01; OR 1.94, 95% CI 1.08–3.49, *P* = 0.03; and OR 5.45, 95% CI 1.77–16.78, *P* = 0.003, respectively).

Twenty-one of the 98 children with CM (21.4%) had both cerebral malaria and severe malarial anemia (CM/SMA). These children were not included in the analysis above, but were compared separately to the children with CM only (*n* = 77) to assess if they differed from this primary group. Children with CM plus SMA had higher group A-EPCR transcript levels (*n* = 21, median, arbitrary units [25^th^ percentile, 75^th^ percentile], 33.8 [10.4–69.5]) than children with CM only (*n* = 77, 14.5 [5.18–34.1], *P* = 0.01, Additional file 1: Table S2), and more specifically, had higher CIDRa1.4/6a transcript levels (*n* = 21, 11.1 [2.85, 20.3]) than children with CM only (*n* = 77, 4.16 [1, 11.7], *P* = 0.02, Additional file 1: Table S2). All other transcript levels were similar between children with CM only and children with CM plus SMA (Additional file 1: Table S2).

### PfEMP1 transcript levels in retinopathy negative CM were intermediate between retinopathy positive CM and SMA

**Fig. 3:**
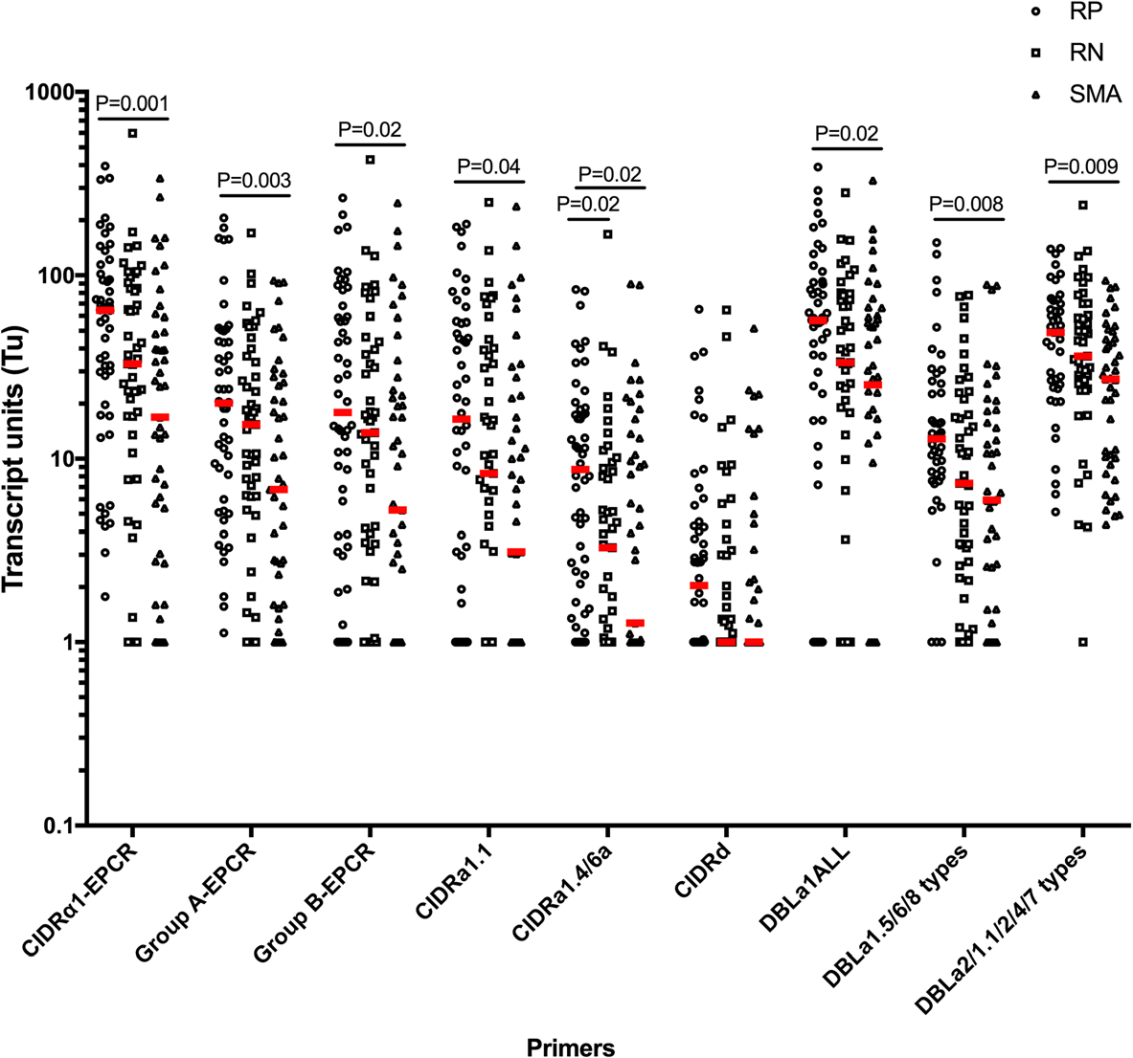
PfEMP1 transcript levels in retinopathy negative CM are intermediate between retinopathy positive CM and SMA, with only CIDRa1.4/6a being higher in retinopathy positive as compared to retinopathy negative CM. Transcript units for CIDRα1-EPCR (sum of [CIDRa1.1-CIDRa1.8b]T_u_-7), group A-EPCR (sum of [CIDRa1.4, CIDRa1.5a, CIDRa1.5b, CIDRa1.6b, and CIDRa1.7]T_u_-4), group B-EPCR (sum of [CIDRa1.1, CIDRa1.8a, and CIDRa1.8b]T_u_-2), CIDRα1.1, CIDRa1.4/6a, DBLa1ALL (all group A PfEMP1), DBLa1.5/6/8 types (group A PfEMP1 typically non-EPCR-binding), CIDRd (group A PfEMP1, non-EPCR-binding), and DBLa2/1.1/2/4/7 (group A PfEMP1, typically EPCR-binding). Transcript units of expression are shown on a logarithmic scale. The horizontal lines in red represent median values. *P* values are estimated by analysis of variance (ANOVA) on log10 transformed arbitrary units followed by Tukey adjustment for multiple comparisons. Retinopathy positive CM (RP CM, *n* = 50), retinopathy negative CM (RN CM, *n* = 47), severe malarial anemia (SMA, *n* = 47)

Next, we compared *var* transcript abundances between RP and RN CM, as well as between RN CM and SMA. In these patients, parasite biomass, as indicated by PfHRP-2 levels, as well as sequestered parasite biomass trended lower in RN CM compared to RP CM (Table 3) when a post hoc adjustment was performed due to the three-way comparison with SMA. When compared side by side, PfHRP-2 levels and sequestered biomass medians are significantly higher in RP than RN (*P* = 0.02 and *P* = 0.04, respectively), as seen in the full cohort published elsewhere [9]. Transcript levels of CIDRα1-EPCR, group A-EPCR, group B-EPCR, CIDRα1.1, CIDRa1.4/6a, DBLa1ALL, DBLa1.5/6/8, and DBLa2/1.1/2/4/7 were all significantly higher in RP CM vs. SMA (*P* < 0.05 for all, Fig. 3). However, only CIDRa1.4/6a transcripts were higher in RP (*n* = 50, 8.74 [2.33, 18.6]) vs. RN CM (*n* = 47, 3.28 [1, 8.88], *P* = 0.02, Fig. 3), and *var* transcript levels were not statistically different between RN CM and SMA (*P* > 0.05 for all), placing RN CM *var* transcript levels consistently between those of children with RP CM and children with SMA (Fig. 3).

**Table 3:** Clinical characteristics of children with retinopathy positive (RP) or retinopathy negative (RN) cerebral malaria (CM) and children with severe malarial anemia (SMA)

	RP (*n* = 50)	RN (*n* = 47)	SMA (*n* = 47)	*P* ^a^
Age (months), median (IQR)	40.1 (29.6–50.2)	42.0 (31.7–59.4)	33.4 (24.9–52.4)	0.23
Sex (male), *n* (%)	29 (58.0)	29 (61.7)	35 (74.5)	0.21
Weight-for-age z-score, mean (SD)	–1.30 (1.26) *n* = 49	–0.92 (1.71)	–1.98 (1.39)	0.002^b^
Hemoglobin (g/dL), mean (SD)	6.34 (2.17)	7.80 (2.21)	3.81 (0.74)	<0.0001^c^
Parasite density (/μL), median (IQR)	100,260 (21,830–415,920) *n* = 48	50,690 (10,780–273,100)	43,880 (11,940–156,040) *n* = 46	0.17
Parasite load (PfHRP-2, ng/mL), median (IQR)	3190 (1418–5222)	2491 (446–3900)	862 (288–2033) *n* = 46	<0.0001^d^
Sequestered biomass (x10^8), median (IQR)	20,880 (11,037–44,350) *n* = 48	15,766 (2450–31,276)	6249 (1303–15,839) *n* = 45	0.0005^e^

^a^Analysis of variance (ANOVA), Tukey post hoc test adjustment for multiple comparisons with log10 transformed values for variables with no normal distribution. Chi-squared test was used for sex, with *P* < 0.017 considered significant to control for multiple comparisons.

^b^In post hoc testing, SMA differed from RN

^c^In post hoc testing, all groups differed from each other

^d^In post hoc testing, SMA differed from RP and RN. For RP vs. RN, *P* = 0.07

^e^In post hoc testing, RP differed from SMA. For RP vs. RN, *P* = 0.08

*IQR* interquartile range, *SD* standard deviation

Due to the difficulties and the expertise needed for indirect ophthalmoscopy, PfHRP-2 levels have been identified as a good predictor of malarial retinopathy. It has previously been shown that PfHRP-2 levels > 1700 ng/mL at enrollment had a 90% sensitivity and 87% specificity in predicting malarial retinopathy [42]. This cutoff yielded a sensitivity for RP vs. RN CM of 72.7% and a specificity of 44.2%. We used this cutoff to redefine two groups within CM: PfHRP-2-high (>1700 pg/mL, *n* = 62) and PfHRP-2-low (≤1700 pg/mL, *n* = 35). Transcript levels of the *var* genes considered in this study did not differ significantly between the PfHRP-2-high and PfHRP-2-low groups (Additional file 1: Table S3). A cutoff of 1392 ng/mL maximized sensitivity and specificity for RP CM compared to RN CM in our study (sensitivity 78.3% and specificity 41.9%). Even when considering our cutoff of 1392 ng/mL, we did not see a difference in *var* transcript levels between PfHRP-2-high and PfHRP-2-low when this cutoff was applied (data not shown). To further assess *var* gene expression in children with RN CM, we compared *var* transcript levels in children with RN CM with PfHRP-2 levels in the lowest quartile to those in children with SMA or AP. PfEMP1 *var* transcripts did not differ significantly between RN children with the lowest quartile of PfHRP-2 levels and children with SMA, another form of severe malaria. Moreover, with the exception of CIDRα1.4 and DBLα2, *var* transcript levels remained significantly higher in RN than in children with AP (Additional file 1: Table S4). Together, these data suggest that *P. falciparum* contributed to disease severity through increased *var* gene expression in almost all of the children with RN CM.

### *P. falciparum* parasites infecting CM children who died had lower transcript levels for *var* genes encoding group A and CIDRα1 domains compared to those for children who survived

**Fig. 4:**
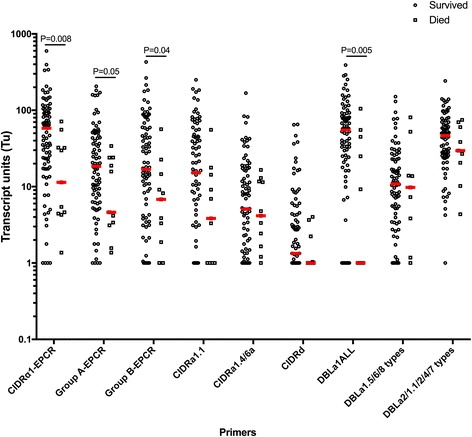
EPCR-binding PfEMP1 transcripts are lower in parasites from patients with cerebral malaria who died. Transcript units for CIDRα1-EPCR (sum of [CIDRa1.1-CIDRa1.8b]T_u_-7), group A-EPCR (sum of [CIDRa1.4, CIDRa1.5a, CIDRa1.5b, CIDRa1.6b, and CIDRa1.7]T_u_-4), group B-EPCR (sum of [CIDRa1.1, CIDRa1.8a, and CIDRa1.8b]T_u_-2), CIDRα1.1, CIDRa1.4/6a, DBLa1ALL (all group A PfEMP1), DBLa1.5/6/8 types (group A PfEMP1 typically non-EPCR-binding), CIDRd (group A PfEMP1, non-EPCR-binding), and DBLa2/1.1/2/4/7 (group A PfEMP1, typically EPCR-binding). Transcript units of expression are shown on a logarithmic scale. The horizontal lines in red represent median values. Medians are compared by Mann-Whitney test. Survived (*n* = 87) and died (*n* = 11)

Transcript levels reported by the DBLa1ALL primers and the summarized levels from primer sets specific to genes encoding CIDRα1 domains were lower in children who died vs. those who survived (*P* ≤ 0.05, Fig. 4). This difference persisted when considering only RP CM children (Additional file 1: Figure S3, trended for group A-EPCR), suggesting that the lower transcript abundance in children with CM who died is not due primarily to misclassification from an additional or different cause of mortality in children with RN CM. Differences also did not appear to be related to bacteremia, because mortality did not differ significantly among the children who had bacteremia vs. those who did not (1/7, 14.3%, for children with bacteremia vs. 8/85, 8.5% for children without bacteremia, *P* = 0.68). Moreover, group A and CIDRa1 *var* transcript levels did not differ between children with vs. without bacteremia (*P* > 0.4 for all, data not shown).

### *var* transcript levels and associations with parasite biomass, sequestered parasite load, or endothelial activation in severe malaria

In all children with severe malaria (children with CM, SMA, or CM/SMA), there was no correlation of transcript levels of any *var* gene group with parasite biomass or sequestered parasite biomass (Table 4).

**Table 4:** Spearman correlations between *var* transcript levels and markers of sequestration and endothelial activation in children with severe malaria (cerebral malaria, severe malarial anemia, or cerebral malaria + severe malarial anemia)

	sICAM-1	sVCAM-1	P-Selectin	E-Selectin	Ang-2	VWF	*Pf*HRP-2	Sequestered load	sEPCR
CIDRα1.1	–0.02 *n* = 133	0.11 *n* = 133	0.07 *n* = 86	0.10 *n* = 133	0.03 *n* = 83	0.07 *n* = 122	–0.04 *n* = 144	0.03 *n* = 128	–0.06 *n* = 127
Group B-EPCR	0.003 *n* = 133	0.11 *n* = 133	0.07 *n* = 86	0.09 *n* = 133	0.06 *n* = 83	0.04 *n* = 122	–0.04 *n* = 144	–0.04 *n* = 141	–0.06 *n* = 127
CIDRα1.4/6a	0.03 *n* = 133	0.03 *n* = 133	0.02 *n* = 86	0.03 *n* = 133	0.08 *n* = 83	0.03 *n* = 122	0.009 *n* = 144	–0.01 *n* = 141	–0.10 *n* = 127
Group A-EPCR	–0.03 *n* = 133	–0.02 *n* = 133	0.02 *n* = 86	–0.06 *n* = 133	0.01 *n* = 83	0.02 *n* = 122	0.02 *n* = 144	0.01 *n* = 141	–0.06 *n* = 127
CIDRα1-EPCR	–0.01 *n* = 133	0.07 *n* = 133	0.05 *n* = 86	0.04 *n* = 133	0.05 *n* = 83	0.04 *n* = 122	–0.02 *n* = 144	–0.02 *n* = 141	–0.07 *n* = 127
CIDR1δ	0.04 *n* = 133	0.02 *n* = 133	0.09 *n* = 86	0.07 *n* = 133	–0.03 *n* = 83	0.006 *n* = 122	–0.04 *n* = 144	–0.004 *n* = 141	–0.14 *n* = 127
DBLα1ALL	–0.06 *n* = 132	0.09 *n* = 132	0.001 *n* = 86	0.04 *n* = 132	0.06 *n* = 83	0.13 *n* = 121	0.002 *n* = 143	–0.005 *n* = 140	–0.12 *n* = 126
DBLα1.5/6/8	0.07 *n* = 123	0.08 *n* = 123	0.22 *n* = 80	0.01 *n* = 123	–0.04 *n* = 80	0.09 *n* = 112	–0.03 *n* = 134	0.006 *n* = 131	–0.12 *n* = 118
DBLα2/1.1/2/4/7	–0.06 *n* = 122	0.25 *n* = 122 ***P*** **= 0.054**	0.13 *n* = 80	0.05 *n* = 122	0.07 *n* = 80	0.13 *n* = 111	–0.07 *n* = 133	–0.07 *n* = 130	–0.03 *n* = 117

*P* values for all correlations in table except DBLα2/1.1/2/4/7 and sVCAM-1 > 0.10.

Among endothelial activation markers, the only comparison that reached close to significance was the association between transcript levels reported by the DBLa2/1.1/2/4/7 primers and sVCAM-1 levels in severe malaria (*P* = 0.054, when adjusted for multiple comparisons, Table 4).

## Discussion

In the present study, we show that children with severe malaria have higher levels of both EPCR-binding group A and DC8 PfEMP1 transcripts than children with asymptomatic parasitemia, that transcript levels of EPCR-binding PfEMP1 are higher in children with CM than SMA, that children with both CM and SMA have higher levels of EPCR-binding group A PfEMP1 transcripts than children with CM alone, and that PfEMP1 transcript levels in RN or PfHRP-2-low CM fall between those in RP CM and those in SMA. Together the findings suggest that not only the presence, but more importantly, the transcript level and therefore the extent of EPCR binding by PfEMP1 may be important in determining the clinical manifestation of SM. A particularly important and novel finding in the current study is the progressive increase in EPCR-binding PfEMP1 expression through the stages of malaria infection and disease, from asymptomatic parasitemia, in which there is very little expression, through SMA to RN CM and RP CM. The finding that EPCR-binding PfEMP1 expression in RN CM falls between that of SMA and RP CM, and far above that in AP, suggests that *P. falciparum* plays a role in the disease process of many children with RN CM, and that RN CM represents a milder disease, a finding consistent with a recent study of clinical manifestations of RP vs. RN CM in this cohort [9].

The findings regarding EPCR-binding PfEMP1 expression are largely consistent with conclusions drawn from two recent studies in Tanzania, showing that CIDRα1 was the only common domain encoded by most prominently expressed *var* transcripts in CM and SMA patients [43], and that higher levels of EPCR-binding PfEMP1 transcripts were associated with increasing symptoms of severity in patients suffering uncomplicated malaria vs. SMA or CM [32]. However, in contrast to the present study, the latter study [32] found no difference in transcript levels of EPCR-binding PfEMP1 between Tanzanian children with CM and SMA, despite application of the same primer set in both studies. The current study had a larger CM group with higher mortality than in the Tanzanian study, and it did not have any mortality in the SMA group. The larger sample size and greater disease severity and mortality in children with CM than SMA in the present study as compared to the Tanzanian study may explain why higher PfEMP1 transcript levels in children with CM as compared to SMA were seen in the present study but not the Tanzanian study [32]. Likewise, expression of other PfEMP1 traits or variants may also account for differences observed between the two populations. In this study, we did not quantify CD36-binding PfEMP1; thus, we cannot infer on the total expression levels of all *vars*, or the proportion of transcripts encoding EPCR-binding PfEMP1 between CM and SMA.

We have found only one other study to date that examines PfEMP1 transcript levels in RP vs. RN CM. In this cohort of Kenyan children, the authors did not find any significant difference in group A, DC8, and CIDRα1.4 transcript levels between RP and RN CM [38], although a higher proportional expression of group A and DC8 compared to group B and C *var* genes was found in patients with RP compared to those with RN [38]. The present study, which uses primers with a better coverage, found only higher levels reported by the CIDRa1.4/6a primers in RP compared to RN CM (Fig. 3). The subset of group A *var* genes targeted by these primers include the so-called domain cassette 13 PfEMP1, which has been shown to often bind both EPCR and ICAM1 [44, 45] and to provide higher binding levels to endothelial cells [44]. Moreover, this PfEMP1 subset has been shown to be more frequently expressed, albeit at lower levels, in patients with CM compared to those with SMA [32, 45]. It is therefore possible that dual EPCR- and ICAM1-binding PfEMP1 account for the higher transcript levels reported by the CIDRa1.4/6a primers in the present study between CM/SMA vs. CM, and RP vs. RN CM patients. Further studies are required to elucidate this hypothesis. We did not assess proportional expression, because transcript levels are not absolute values, and no study captures 100% of *var* diversity in a patient, so proportional values can be strongly influenced by outlier values.

The present study provides two important additional pieces of information: RN CM transcript levels fall between those of two forms of severe malaria, RP CM and SMA, and transcript levels are similar in children above and below a proposed PfHRP-2 cutoff level that would indicate “true” CM. Together the findings provide evidence suggesting that *P. falciparum* sequestration via PfEMP1 plays a role in the development of RN CM. The finding that PfEMP1 expression did not differ between those with levels above and below a suggested cutoff for PfHRP-2 levels to define “true” CM [42] also suggests that PfHRP-2 levels may be less useful than hoped in distinguishing “true” CM from coma due to other causes with incidental parasitemia. Assessment of *var* transcript levels in the field is unlikely to ever be a practical diagnostic tool, but it could be very useful in future research studies of CM for attributing coma to *P. falciparum* or another cause. Retinopathy could also be occurring at levels not detectable by standard funduscopic exam, and our study medical officers may have occasionally missed retinal findings that would be seen by an ophthalmologist, but having received training and validation of testing mid-study from highly experienced ophthalmologists, they likely represent a “gold standard” for field ophthalmoscopy testing. Newer technologies for assessing retinopathy with camera and/or radiologic imaging may provide better understanding of the extent to which “subclinical” retinopathy is occurring, but these methods are also likely to remain limited to research.

Interestingly, transcript levels reported by the DBLa1ALL primers and the summarized levels from primer sets specific to genes encoding CIDRα1 domains were lower in children who died, despite their having higher PfHRP-2 levels as compared to survivors. This remained true when analysis was restricted to RP CM, confirming that death was most likely caused by *P. falciparum* infection. A similar trend towards lower *var* transcript abundance in children with CM who died was observed in one previous study [31] but not in a more recent study [32]. These inconsistencies may reflect the complexities of the disease at the end of life complicated by the limited number of samples for children who died. DBLα1ALL and CIDRα1 transcript levels were particularly low for around half of the children who died. In these children, a transcript level above baseline was picked up by the DBLa2/1.1/2/4/7 primers, suggesting that either rare group A or CD36-binding PfEMP1 was expressed, and possibly associated with death in these children. Possible biological reasons for an altered *var* profile compared to that for surviving SM patients include that a particularly adverse host response to infection, unrelated to or even allowing diverse PfEMP1 phenotypes, led to death. Even though we found the same results for RP children, it cannot be completely ruled out that another co-infection that increases the risk of mortality in SM, such as bacteremia [46], could be contributing to death in those children with CM who have low group A and CIDRa1 transcript levels. However, in the present study we did not find an association between PfEMP1 transcript levels and the presence of bacteremia in children with CM. Deeper characterization of the *var* transcripts in these patients, as well as thorough testing for other co-infections, may offer clues as to the reason for the unexpected finding of lower group A and DC8 *var* transcript levels in children with CM who died.

The present study does not provide clear information on the clinical relevance of rosetting. While most rosetting PfEMP1 types are group A and carry DBLα1.5/6/8 domains [40, 41], it is still unclear if such domains, or a specific subset of these, consistently confer rosetting. In future studies, we plan to assess transcript levels of groups B and C CD36-binding PfEMP1, which have shown to be similar [28] or higher [47] in AP as compared to uncomplicated malaria or SM in prior studies. We did not enroll children with uncomplicated malaria in this study, and assessment of PfEMP1 transcript levels in this group, who represent another important comparison group of malaria without severe manifestations, will be important for future studies. However, the AP group in this study had no history of prior SM, and only one experienced SM over the 2 years of follow-up, despite presumably similar malaria exposure (since they lived in the same extended households as children with SM). As a result, AP represents an important comparison group, since parasites from patients with uncomplicated malaria could still express some of the domains associated with SM, even though the children present with uncomplicated malaria, because they are protected from development of SM by early treatment.

Only results reported by DBLa2/1.1/2/4/7 primers were weakly but not significantly associated with endothelial activation (specifically increased sVCAM-1 levels) in SM. This suggests that at this stage of the disease, pathways that lead to sequestered parasite load and endothelial activation are more complex than simply PfEMP1 binding to host receptors. In the current study, none of the EPCR-binding PfEMP1 transcript levels were associated with plasma levels of sEPCR, suggesting that binding of PfEMP1 to EPCR might prevent shedding of EPCR in an inflammatory context. This potential mechanism would be interesting to explore in vitro with parasite strains that bind specifically to EPCR.

## Conclusions

The present study suggests that EPCR-binding PfEMP1 expression is important in the development of severe malaria, and that increased EPCR-binding PfEMP1 expression is associated with progressively more severe disease. The presence of high levels of EPCR-binding PfEMP1 transcripts in RN and/or PfHRP-2-low CM further suggests that *P. falciparum* is playing a role in RN CM. The study provides the strongest evidence to date that *P. falciparum*, via PfEMP1, is involved in the pathogenesis of both RP and RN/PfHRP-2-low CM, and thus provides further support for PfEMP1, and in particular EPCR-binding PfEMP1, as a target for interventions to prevent severe malaria.

## Additional file

Additional file 1:
**Table S1.** Comparison of clinical characteristics between children who had samples for *var* qRT-PCR vs. those who did not. **Table S2.** Transcript abundance of *var* domains in children with cerebral malaria vs. those who have both CM and SMA. **Table S3.** Transcript levels of *var* domains in children with cerebral malaria (CM) who had PfHRP-2 levels > 1700 pg/mL (high) or (≤1700 pg/mL (low). **Table S4.** Transcript levels of *var* domains in children with retinopathy negative cerebral malaria with the lowest quartile of PfHRP-2 levels vs. severe malarial anemia and asymptomatic parasitemia. **Figure S1.** In silico characterization of primers used in the present study. **Figure S2.** Detection of housekeeping genes in parasites infecting asymptomatic controls. **Figure S3.** DBLa1ALL, group A-EPCR, and CIDRa1-EPCR transcript levels are lower in parasites from retinopathy positive patients who died. (DOCX 7045 kb)

## Abbreviations

CM: Cerebral malaria
EPCR: Endothelial protein C receptor
PfEMP1: *Plasmodium falciparum* erythrocyte membrane protein 1
RN: Retinopathy negative
RP: Retinopathy positive
SMA: Severe malarial anemia
