# Supplementary material for: Plasmodium falciparum EPCR-binding PfEMP1 expression increases with malaria disease severity and is elevated in retinopathy negative cerebral malaria
Source: BMC Med. 2017 Oct 13;15:183. doi: 10.1186/s12916-017-0945-y (PMC5639490; doi:10.1186/s12916-017-0945-y)
Supplement: Additional file 1: — Table S1. Comparison of clinical characteristics between children who had samples for var qRT-PCR vs. those who did not. Table S2. Transcript abundance of var domains in children with cerebral malaria vs. those who have both CM and SMA. Table S3. Transcript levels of var domains in children with cerebral malaria (CM) who had PfHRP-2 levels > 1700 pg/mL (high) or (≤1700 pg/mL (low). Table S4. Transcript levels of var domains in children with retinopathy negative cerebral malaria with the lowest quartile of PfHRP-2 levels vs. severe malarial anemia and asymptomatic parasitemia. Figure S1. In silico characterization of primers used in the present study. Figure S2. Detection of housekeeping genes in parasites infecting asymptomatic controls. Figure S3. DBLa1ALL, group A-EPCR, and CIDRa1-EPCR transcript levels are lower in parasites from retinopathy positive patients who died. (DOCX 7045 kb) [file 12916_2017_945_MOESM1_ESM.docx]

*Plasmodium falciparum* EPCR-binding PfEMP1 expression increases with malaria disease severity and is elevated in retinopathy negative cerebral malaria

Estela Shabani, Benjamin Hanisch, Robert O. Opoka, Paul Bangirana, Thomas Lavstsen and Chandy C. John

|  | **CM** | | | **SMA** | | | **AP** | | |
| --- | --- | --- | --- | --- | --- | --- | --- | --- | --- |
|  | Tested  N=98 | Not tested  N=171 | P^a^ | Tested  N=47 | Not tested  N=185 | P^a^ | Tested  N=14 | Not tested  N=18 | P^a^ |
| Age (months), median (IQR) | 41.5  (30.9-54.6) | 42.0  (30.0-60.1) | 0.88 | 33.4 (24.9-52.4) | 33.8 (24.8-50.7) | 0.98 | 48.5  (31.0-71.0) | 46.4  (33.4-60.0) | 0.72 |
| Sex (male), n (%) | 59 (60.2) | 100 (58.5) | 0.78 | 35 (74.5) | 105 (56.8) | 0.03 | 7 (50.0) | 10 (55.6) | 0.76 |
| Weight for age z-score, mean (SD) | -1.11 (1.49)  n=97 | -1.28 (1.23)  n=169 | 0.31 | -1.98 (1.39) | -1.57 (1.46)  n=182 | 0.08 | -0.31 (1.17) | -1.06 (1.01) | 0.06 |
| Hemoglobin (g/dL), mean (SD) | 7.07 (2.30) | 6.91 (2.30) | 0.58 | 3.81 (0.74) | 3.72 (0.90) | 0.52 | 11.2 (2.15) | 11.5 (1.26)  n=15 | 0.65 |
| Parasite density (/μl), median (IQR) | 67010  (18030-347010) n=96 | 39990 (9420-201060)  n=166 | 0.04 | 43880  (11940-156040) n=46 | 34060 (10040-137580) | 0.36 | 2170  (520-11880) | 1060  (720-4800) | 0.70 |
| Parasite load (*Pf*HRP-2, ng/ml), median (IQR) | 2648  (883-5150) | 2917  (1127-5675) | 0.38 | 862 (288-2033)  n=46 | 959 (382-2790)  n=183 | 0.23 | 88.8 (4.80-158)  n=13 | 4.80 (4.80-52.8)  n=17 | 0.13 |
| Sequestered biomass (x10^8), median (IQR) | 17928  (5323-39891)  n=96 | 22187  (8267-43094)  n=166 | 0.36 | 6249  (1303-15839)  n=45 | 7078 (2187-19823)  n=183 | 0.25 | 469  (0-1309)  n=13 | 0 (0-272)  n=14 | 0.24 |
| Mortality, n (%) | 11 (11.2) | 23 (13.5) | 0.60 | 0 | 0 |  | 0 | 0 |  |

**Additional file 1: Table S1. Comparison of clinical characteristics between children that had samples for *var* qRT-PCR vs. those that did not**

^a^ Continuous variables were compared by ANOVA when normally distributed and by Wilcoxon rank-sum (Mann-Whitney) test if not normally distributed. Categorical variables were compared by χ^2^test.

**Additional file 1: Table S2. Transcript abundance of *var* domains in children with cerebral malaria vs. those that have both CM and SMA**

| Primers | CM and SMA  (n=21) | CM  (n=77) | P^a^ |
| --- | --- | --- | --- |
| CIDRa1.1 | 14.3 (1-55.6) | 10.4 (1-43.4) | 0.72 |
| Group B-EPCR binders | 15.1 (5.87-56.6) | 15.2 (3.80-48.6) | 0.51 |
| CIDRa1.4/6a | 11.1 (2.85-20.3) | 4.16 (1-11.7) | 0.02 |
| Group A-EPCR binders | 33.8 (10.4-69.5) | 14.5 (5.18-34.1) | 0.01 |
| CIDRα1-EPCR binders | 66.7 (30.2-136) | 36.9 (17.0-91.1) | 0.11 |
| CIDRd | 2.73 (1-6.05) | 1 (1-3.44) | 0.12 |
| DBLa1ALL | 73.7 (25.0-140) | 40.5 (9.88-77.6) | 0.09 |
| DBLa1.5/6/8 types | 13.7 (8.23-31.1)  n=18 | 10.4 (3.45-22.6)  n=73 | 0.11 |
| DBLa2/1.1/2/4/7 types | 48.6 (27.3-98.1)  n=18 | 43.5 (24.5-60.5)  n=73 | 0.27 |

^a^ Median transcript levels were compared using Mann-Whitney U test

**Additional file 1: Table S3. Transcript levels of *var* domains in children with retinopathy negative cerebral malaria with the lowest quartile of PfHRP-2 levels vs. severe malarial anemia and asymptomatic parasitemia**

| **Primers** | **RN with PfHRP2 in lowest quartile (n=11)** | **SMA**  **(n=47)** | **AP**  **(n=14)** | **P**  **RN vs. SMA** | **P**  **RN vs. AP** |
| --- | --- | --- | --- | --- | --- |
| Parasite load (*Pf*HRP-2, ng/ml), median (IQR) | 266 (166-321) | 862 (288-2033)  n=46 | 88.8 (4.8-158)  n=13 | 0.006 | 0.01 |
| CIDRa1.1 | 6.72 (1-33.0) | 3.11 (1-21.4) | 1 (1-1) | 0.58 | 0.004 |
| Group B-EPCR binders | 10.4 (1-37.2) | 5.24 (1-23.7) | 1 (1-1.23) | 0.66 | 0.007 |
| CIDRa1.4/6a | 1 (1-8.00) | 1.27 (1-13.0) | 1 (1-1) | 0.31 | 0.07 |
| Group A-EPCR binders | 10.5 (1-18.8) | 6.78 (1.53-26.1) | 1 (1-1.06) | 0.67 | 0.02 |
| CIDRα1-EPCR binders | 23.3 (1-46.7) | 16.9 (2.77-49.4) | 1 (1-1.99) | 0.73 | 0.02 |
| CIDRd | 1 (1-1.79) | 1 (1-2.21) | 1(1-1) | 0.74 | 0.08 |
| DBLa1ALL | 1 (1-24.3) | 25.4 (1-59.1)  n=46 | 1(1-1) | 0.08 | 0.055 |
| DBLa1.5/6/8 types | 7.34 (1.20-23.0) | 5.95 (1.39-17.0)  n=44 | 1(1-1)  n=10 | 0.77 | 0.003 |
| DBLa2/1.1/2/4/7 types | 30.8 (8.17-60.5) | 27.3 (10.1-45.7)  n=43 | 12.9  (6.18-19.3)  n=10 | 0.34 | 0.12 |

**Additional file 1: Table S3. Transcript levels of *var* domains in children with cerebral malaria (CM) who had PfHRP2 levels ≥ 1700ng/ml (high) or <1700ng/ml (low)**

| Primers | *Pf*HRP-2-high CM  (n=62) | *Pf*HRP-2-low CM  (n=35) | P^a^ |
| --- | --- | --- | --- |
| CIDRa1.1 | 13.0 (1-43.3) | 10.9 (1-48.3) | 0.99 |
| Group B-EPCR binders | 14.8 (3.80-46.2) | 16.6 (3.44-58.6) | 0.95 |
| CIDRa1.4/6a | 4.76 (1.35-16.6) | 5.08 (1-12.7) | 0.45 |
| Group A-EPCR binders | 18.2 (5.24-36.6) | 15.4 (7.14-43.9) | 0.76 |
| CIDRα1-EPCR binders | 38.6 (17.0-84.6) | 58.1 (23.3-102) | 0.48 |
| CIDRd | 1.34 (1-4.02) | 1 (1-5.57) | 0.55 |
| DBLa1ALL | 53.8 (20.8-78.0) | 36.9 (1-107) | 0.62 |
| DBLa1.5/6/8 types | 10.8 (5.21-26.6)  n=55 | 11.5 (2.75-22.9) | 0.70 |
| DBLa2/1.1/2/4/7 types | 43.5 (23.6-69.8)  n=55 | 49.1 (28.4-70.2) | 0.42 |

^a^ Median transcript levels were compared using Mann-Whitney U test

**Additional file 1: Figure S1. In siico characterization of primers used in the present study**

**
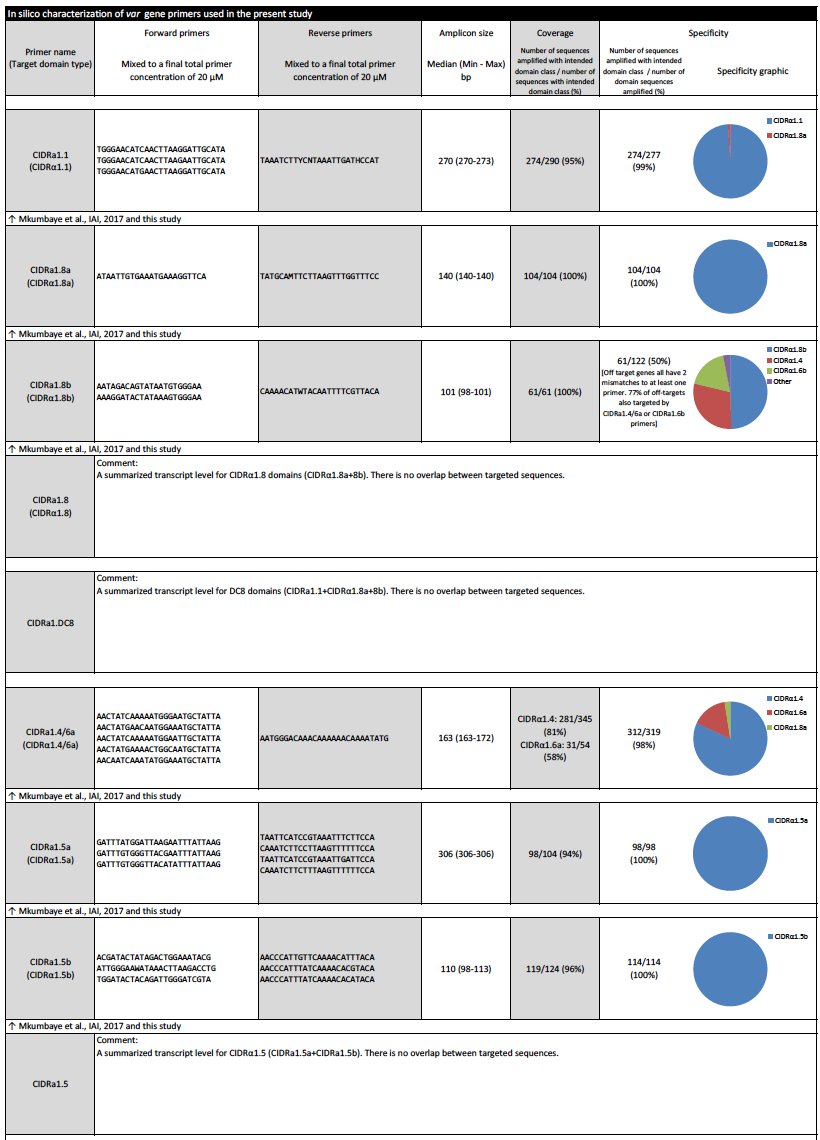
**

**
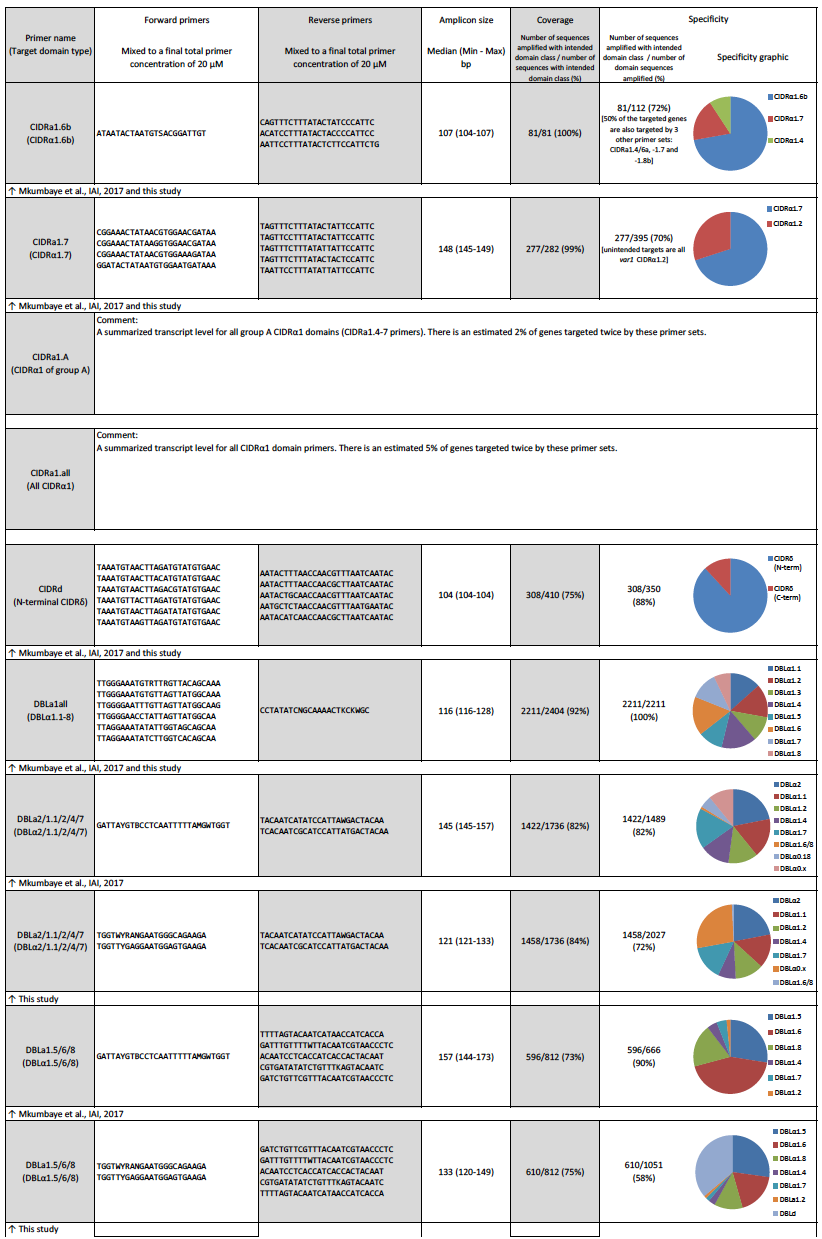
**

**
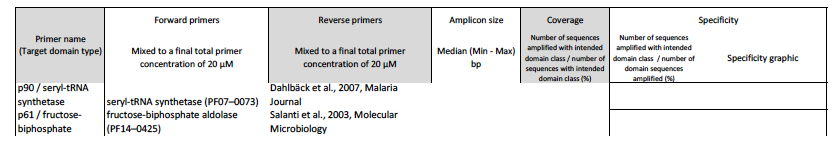
**

**
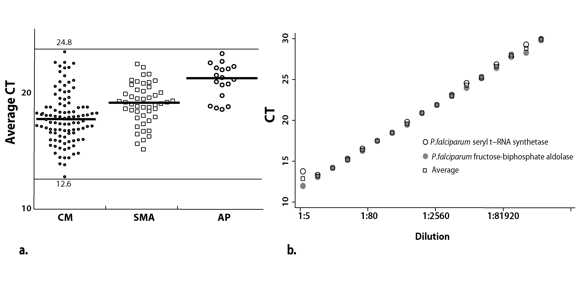
Additional file 1: Figure S2. Detection of housekeeping genes in parasites infecting asymptomatic controls.** (a) Median comparison of average Ct values for the two housekeeping genes (seryl t RNA synthetase and fructose-bisphosphate aldolase) between disease groups. Average Ct values are shown on a logarithmic scale. The horizontal line represents median values. b) Standard curves for both housekeeping genes and the average based on dilutions of 3D7 gDNA. Cerebral malaria (CM), severe malarial anemia (SMA) and asymptomatic parasitemia (AP).

| **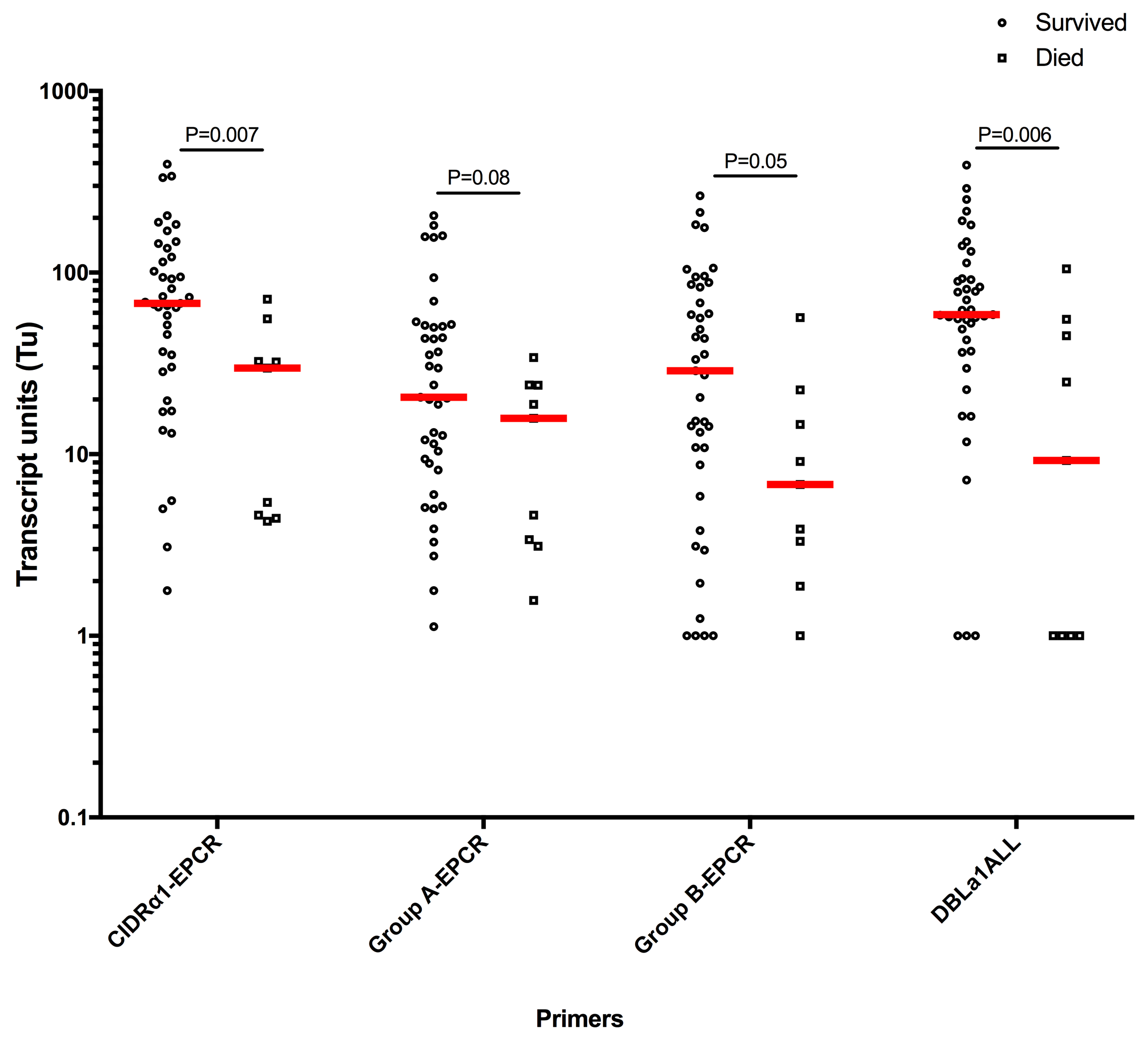** |
| --- |

**Additional file 1: Figure S3. DBLa1ALL, group A-EPCR and CIDR**a**1-EPCR transcripts levels are lower in parasites from retinopathy positive patients that died.** Transcript unit values for CIDRα1-EPCR (sum of [CIDRa1.1-CIDRa1.8b]T_u_-7) , Group A-EPCR (sum of [CIDRa1.4, CIDRa1.5a, CIDRa1.5b, CIDRa1.6b and CIDRa1.7]T_u_-4), Group B-EPCR (sum of [CIDRa1.1, CIDRa1.8a and CIDRa1.8b]T_u_-2) and DBLa1ALL (all group A PfEMP1). Medians are compared by Mann-Whitney test. Only retinopathy positive cerebral malaria cases are presented in this figure. Survived (n=41), died (n=9).
